# Simulation Study on the Integration of Health Traits in Horse Breeding Programs

**DOI:** 10.3390/ani10071153

**Published:** 2020-07-07

**Authors:** Lisa Büttgen, Johannes Geibel, Henner Simianer, Torsten Pook

**Affiliations:** Department of Animal Sciences, Center for Integrated Breeding Research, University of Goettingen, Albrecht-Thaer-Weg 3, 37075 Goettingen, Germany; johannes.geibel@uni-goettingen.de (J.G.); hsimian@gwdg.de (H.S.); torsten.pook@uni-goettingen.de (T.P.)

**Keywords:** animal breeding, selection, riding horses, OCD, MoBPS, animal welfare, functional traits

## Abstract

**Simple Summary:**

The disease osteochondrosis dissecans (OCD) is an important health-related trait in horse breeding. This study aimed at finding a breeding strategy to reduce the occurrence of OCD without affecting the riding horse performance traits substantially. Therefore, a lifelike simulation of different possible ways to include this trait in a horse breeding program was performed. Effective strategies can be the exclusion of affected animals from breeding as well as the selection based on a breeding value estimation which considered OCD susceptibility together with riding horse performance traits in an index. A reduction in the frequency of occurrence of OCD was found to be linked with a slight decrease in the breeding values for the riding horse characteristics.

**Abstract:**

Osteochondrosis dissecans (OCD) is a degenerative disease of the cartilage leading to osseous fragments in the joints. It is important in horse breeding both from an animal welfare and an economic perspective. To study adequate breeding strategies to reduce OCD prevalence, a lifelike simulation of the breeding program of German Warmblood horses was performed with the R package MoBPS. We simulated complex breeding schemes of riding horses with different selection steps and realistic age structure, mimicking the German situation. As an example, osseous fragments in fetlock and hock joints were considered. Different scenarios, either using threshold selection, index selection or genomic index selection, respectively, were compared regarding their impact on health and performance traits. A rigorous threshold selection as well as the integration of OCD in a selection index at the stage of stallion licensing and chosen frequency of use in breeding cases on a selection index that includes breeding values for OCD traits performed best on a comparable level. Simply integrating OCD in this breeding value was less effective in terms of OCD reduction. Scenarios with a higher reduction of OCD also showed a slightly reduced improvement in the riding horse performance traits.

## 1. Introduction

Health traits are of central importance in horse breeding. Breeding animals that are healthy is primarily desirable for animal welfare reasons. For breeders and subsequent users, economic considerations are closely linked to this goal, as only healthy animals can perform consistently well over a long time and thus generate profitable sales revenues [1]. An example of a disease that is particularly relevant in riding horse breeding is osteochondrosis dissecans (OCD). The disturbance of the endochondral ossification in young horses (Osteochondrosis) can lead to detachment of osteochondral fragments (OCD) [2], which can be indicated by genetic and biochemical disruptions, ischemia and disturbance of chondrocyte biogenesis leading to failure in cartilage canals [3]. Typically, OCD is diagnosed by clinical inspection and radiographic imaging, which is also used for phenotyping when considering OCD in a breeding program. [2] According to [4], OCD is the most common orthopedic finding in horses that requires surgery. Both environmental and genetic factors play a role in the development of OCD. Heritability estimates of h^2^ = 0.162 [5] and h^2^ = 0.189 [6] were found for OCD prevalence in the fetlock joint, and heritability estimates of h^2^ = 0.369 [6] and h^2^ = 0.462 [5] in the hock joint for Hanoverian Warmblood horses.

The performance traits of riding horses bred for show jumping and dressage sports show predominantly slightly favorable correlations with the amount of radiological findings at riding horse auctions [7]. However, a threshold selection for the health traits before the actual selection for performance traits can lead to a situation where the number of candidates still available for selection afterwards is reduced and thus the selection intensity for performance traits is impaired. Depending on which phenotypic characteristic is used as the selection threshold, this can lead to a significant reduction in the number of selection candidates, as, for example, 31.6% of all auction horses in the period from 1991 to 1998 had at least one diagnosed bone fragment [8]. Another possibility for targeted breeding against OCD is to include this trait in the selection index of the breeding values for approved stallions or in a breeding value estimation on the stage of stallion licensing [9]. Moreover a genomic breeding value estimation for OCD can be considered since the genetic background of OCD has been well studied in German Warmblood horses and has been shown to be of complex nature [10,11,12]. Although efforts are being made to create a comprehensive health database for horses in order to be able to use this data in later breeding decisions, there is still considerable potential to improve the breeding for health traits [13].

The aim of the present study was to compare different strategies of recording OCD in two different joints as exemplary health traits and the selection based on these traits with regard to their breeding efficiency, as well as the impact of these strategies for the riding horse performance traits. For this purpose, a complex riding horse breeding program mimicking German riding horse breeding was simulated in order to derive recommendations for practical breeding considerations.

## 2. Materials and Methods

The simulation of the breeding programs was carried out in the graphical user interface (https://mobps.de), which is connected to the R-package MoBPS (Modular Breeding Program Simulator) [14]. The R-package MoBPS, which is based on stochastic simulations, allows complex breeding programs to be modeled and different options of trait recording and selection to be compared. Reproduction, selection and breeding value estimation (BVE), as well as an age structure, were considered in the simulations.

The basic breeding program was built according to the breeding program of German riding horse breeding. In the simulation, a riding horse population of the size of the German Hanoverian population—which, with 15,658 breeding mares and 540 breeding stallions, is the German warmblood breed with the most registered breeding animals [15]—was considered as representative for German warmblood horse breeds.

Foal shows, studbook registration, mare performance test, 14-day performance test for stallions and 50-day performance test were included in the simulated breeding program. For the foal shows the traits type, exterior and movement were simulated. For all other stages, the traits walk, trot and canter were considered as well as for the mare performance test, the 14-day performance test for stallions and the 50-day performance test for stallions, additionally the traits rideability and free-jumping were also considered. In addition, course jumping was considered in the 50-day performance test. Cohort sizes were derived from animal numbers for registered stallions, broodmares and foals, as well as the numbers of breeding animals that have participated in certain selection events from the data published by the German Equestrian Federation (FN) [15].

**Figure 1:**
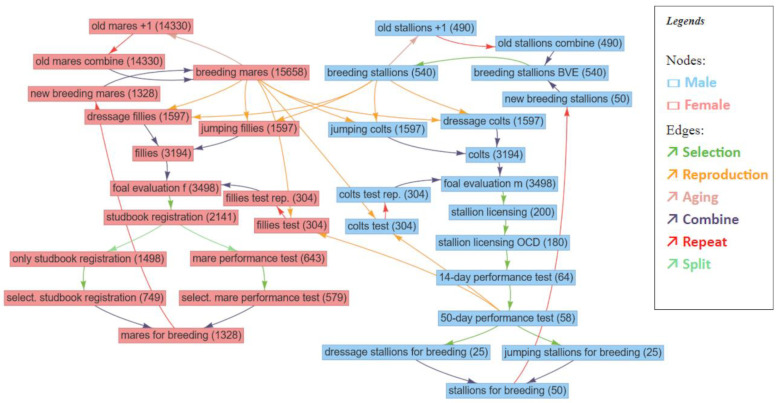
Schematic illustration of the simulated breeding program from the user interface of MoBPS. Numbers in brackets indicate the maximum size of the cohort.

A schematic overview of the simulated breeding program is given in Figure 1. As an initial step, fillies and colts were assessed in a foal show. For mares, the studbook registration and subsequent mare performance tests for about one third of the animals was simulated. For the stallions, the selection steps were represented by simulating stallion licensing, the 14-day performance test and the 50-day performance test. For the latter it was assumed that half of the breeding stallions were selected according to dressage traits and the other half according to jumping traits. In addition, a one-year breeding assignment after the successful assessment test was simulated before the 50-day performance test. It was assumed that the combination of the 14-day performance test and the 50-day performance test can be regarded as equivalent to all ten other possible paths to final breeding approval for German Warmblood stallions [16]. The breeding stallions cohort consisted of the former breeding stallions aged by one year and the new breeding stallions selected in the 50-day performance test.

For each animal, a total of 50.000 underlying single nucleotide polymorphisms (SNPs) were simulated. This corresponds to the size of the Illumina EquineSNP50 BeadChip [17]. All performance traits were simulated as quantitative traits with purely additive underlying quantitative trait loci (QTLs). Traits were simulated with heritability, phenotypic standard deviation (Table 1) [18,19] as well as genetic and phenotypic correlation between traits according to [1,2,7,8,9] (c.f. Table S1). For entries of the correlation matrix with no reliable estimate in the literature, plausible values were sensibly added assuming genetic correlations to be similar to the correlations to the same trait in other selection steps and unknown residual correlations between different selection steps to be close to zero (c.f. correlation matrices in File S1).

Furthermore, the bone health traits “bone fragments in the fetlock joints” (OCD-FJ) and “bone fragments in the hock joints” (OCD-HJ) were simulated to have binary phenotypes, coding the presence/absence of at least one fragment in the respective joints, based on quantitative genomic values to control OCD probability for each individual animal. Furthermore, the trait height at withers was included in the simulation, but not used in the selection index. The heritability estimates of 0.189 for the fetlock joints and 0.369 for the hock joints were taken from Stock et al. [6]. The genetic correlation between these two traits was moderately negative (Table 2) [6]. The correlations between performance traits and occurrence of OCD were taken from Stock and Distl (Table 3) [7]. They are predominantly slightly negative and therefore are in a favorable direction from a breeding point of view.

The selection was based on a selection index with equal weights on all performance traits that were already collected in each respective step. Selection on the female side was assumed to be done on phenotypes and for males based on estimated breeding values, as breeding value estimation for German Warmblood horses mainly focuses on stallions and only includes a very limited number of mares. For breeding value estimation, the classical best linear unbiased prediction (BLUP) animal model [20] based on a pedigree with a depth of seven generations was used. Although MoBPS also offers the possibility to estimate the variance components for the breeding value estimation from the given data, variance components were assumed to be known to save computing time in the simulation. Frequency of the use of stallions for breeding was modelled to be dependent on their estimated breeding value such that the best stallions are aimed to be used about 100 times more frequently than the worst selected stallion. Note that in the actual simulations breeding stallions were used 73 times at the most. In all selection levels the traits considered were scaled according to the standard deviation of the estimated breeding values. To simulate a realistic age distribution of the breeding animals, individuals at each class were assigned a probability to be no longer available for breeding. The used age distribution was derived based on the age difference between all stallions licensed for German riding horse associations between 1990 and 2016 and their parents, and was assumed to have no underlying genetic influences. Furthermore, it was assumed that the generation intervals estimated for the stallions’ sires and stallions’ dams correspond approximately to those of the mares’ sires and mares’ dams.

To simulate the effects of a variety of options to include bone health traits into the selection steps and various recording schemes for them, the nine scenarios given in Table 4 were considered. A reference without selection against OCD was compared to four different scenarios (ThreshSel1, ThreshSel2, ThreshSelFJ, ThreshSelHJ) with threshold selection against one or both traits before stallion licensing. Next, two scenarios (IndexBVE1, IndexBVE2) that integrated the health traits in the breeding value estimation for approved stallions using varying numbers of phenotyped animals were considered. Two further scenarios which additionally performed a breeding value estimation at the stage of stallion licensing that included the health traits (IndexLicensing1, IndexLicensing2) were simulated. The reference scenario can be found as a template at www.mobps.de and input files for all scenarios in the graphical interface are given in Supplementary File S1. 

In all scenarios apart from the reference scenarios, phenotypes for the traits bone fragments in the fetlock joint and bone fragments in the hock joint were collected at the stallion licensing for all preselected candidates as binary traits, coding presence/absence of OCD in the respective joint. In the scenarios IndexBVE2 and IndexLicensing2, it was assumed that bone fragments were recorded for 50% of all other horses. This should represent the case of X-ray images from pre-purchase inspections being collected in a health database. These traits were then each considered in the breeding value estimation with the same weight as a performance trait.

Additionally, the scenarios “Reference”, “IndexBVE2”, “IndexLicensing1” and “IndexLicensing2” were modeled using genomic BVE (ReferenceG, IndexBVEG2, IndexLicensingG1 and IndexLicensingG2) via single-step genomic best linear unbiased prediction (GBLUP) [21]. Here it was assumed that in addition to the phenotypes, SNP genotypes were available for all stallions presented for licensing. All other parameters of the breeding program remained constant.

In order to report the performance traits, the standardized breeding values of the characteristics walk, trot, canter, rideability, free jumping and course jumping of the stallion performance test were combined in an index with equal weighting. For each scenario, 25 simulation runs were performed and reported results represent average values from these independent runs. For the traits OCD in fetlock joint and OCD in hock joint regression lines based on linear regression of the mean values of the 25 replicated simulations and their 95% confidence intervals were added to the plots. Furthermore, the accuracy of the breeding value estimation for the cohort of breeding stallions was calculated by assessing the correlation of the underlying true genomic value and the estimated breeding value.

## 3. Results

**Figure 2:**
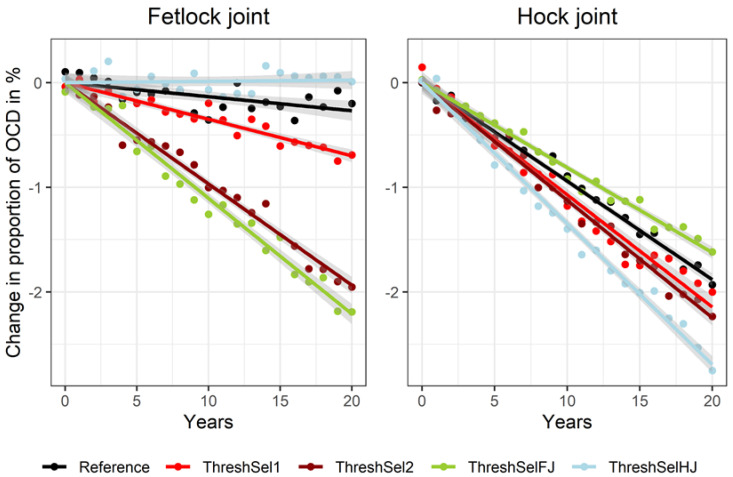
Proportion of unselected offspring with OCD in fetlock joints and hock joints for a reference scenario and for four different scenarios using threshold selection against OCD.

When comparing resulting frequencies of OCD occurrence across the scenarios, results for the two considered joints were rather different. Even though there was no direct index weight on OCD in the hock joint, the reference scenario lead to a substantial improvement within 20 years of breeding (−1.9% occurrence), whereas OCD in the fetlock joint remained basically constant (−0.3% occurrence; Figure 2). 

A threshold selection, discarding horses with OCD in fetlock or hock joints (ThreshSel2) showed a substantial decrease in the occurrence of OCD of about −2.0% in the fetlock joint and about −2.2% in the hock joint within 20 years. In the threshold selection, only stallions showing OCD in both joints were excluded from breeding (ThreshSel1), the result for the decrease in OCD occurrence in the hock joint was the same as in the previous scenario (ThreshSel2), but the decrease in the fetlock joint was much lower (0.9% within 20 years). Selecting only against OCD in fetlock joint (ThreshSelFJ) led to a comparable reduction of OCD frequency for this joint (−2.1%) as in the ThreshSel2 scenario, but for the hock joint the reduction of OCD was less than in the Reference (−1.5%). When selecting only against OCD in the hock joint (ThreshSelHJ), the highest reduction of OCD in the hock joint (−2.7%) among the threshold selection scenarios was observed, but led to an increase of OCD in the fetlock joint (+0.2%; Figure 2).

**Figure 3:**
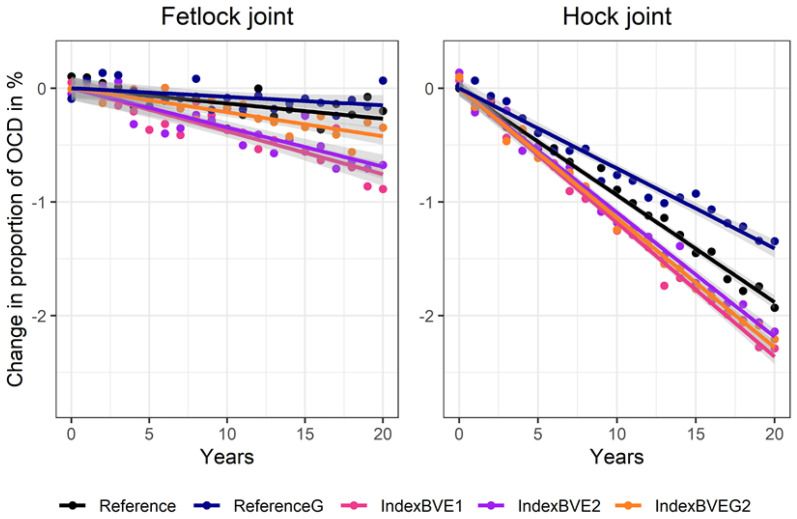
Proportion of unselected offspring with OCD in fetlock joints and hock joints for two reference scenarios and for three different scenarios including OCD in the breeding value estimation.

The inclusion of OCD traits in the selection index to determine the frequency of breeding use of the stallions (IndexBVE1) had similar impact (−0.9 fetlock joint, −2.3% hock joint) as the threshold selection when removing stallions with both joints affected (ThreshSel1; Figure 3). Even though the accuracy of the breeding value estimation increased considerably (Table 5) in the second scenario (IndexBVE2), as substantially more phenotypes were generated, the resulting genomic gains were rather similar (Figure 3). For OCD in the fetlock joint, the accuracy increased from 0.34 to 0.53, and for OCD in the hock joint from 0.35 to 0.47 (Table 5). When additionally generating genomic data for all licensed stallions and subsequently using single-step GBLUP for breeding value estimation, selection gains were slightly increased, but the differences were never greater than 0.02. Overall results in terms of reduction of OCD for genomic selection were very similar to pedigree-based selection with slightly greater differences between genomic scenarios and their reference (Figure 3).

**Figure 4:**
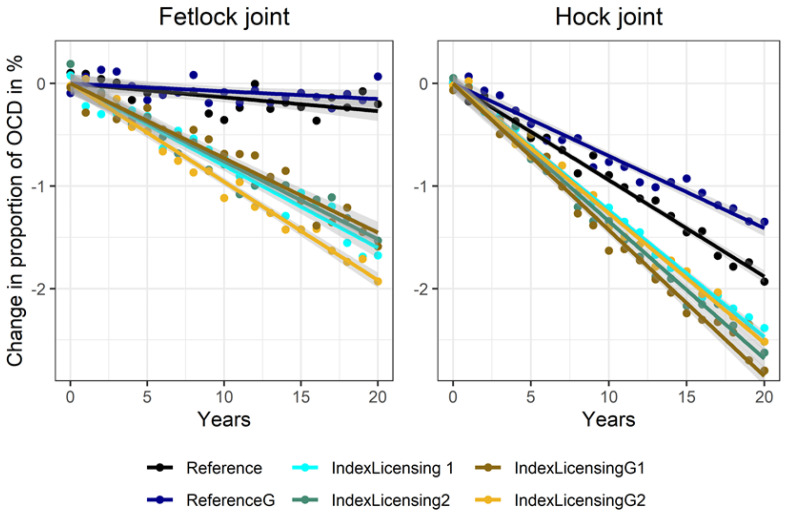
Proportion of unselected offspring with OCD in fetlock joints and hock joints for two reference scenarios and for four different scenarios including OCD in the breeding value estimation and in a selection index at the stage of stallion licensing.

When additionally performing a selection step based on a selection index that considers OCD and the riding horse performance traits at the stage of stallion licensing (IndexLicensing1 and IndexLicensing2), improvements were on a comparable level to those obtained when using the best performing threshold selection (−1.6% and −1.6% in fetlock joint; −2.4% and −2.6% in the hock joint). The reduction of OCD frequency over time was very similar when using genomic breeding value estimation (IndexLicensingG1 and IndexLicensingG2). Note, however, that differences to the respective reference scenarios were again substantially larger (Figure 4).

**Figure 5:**
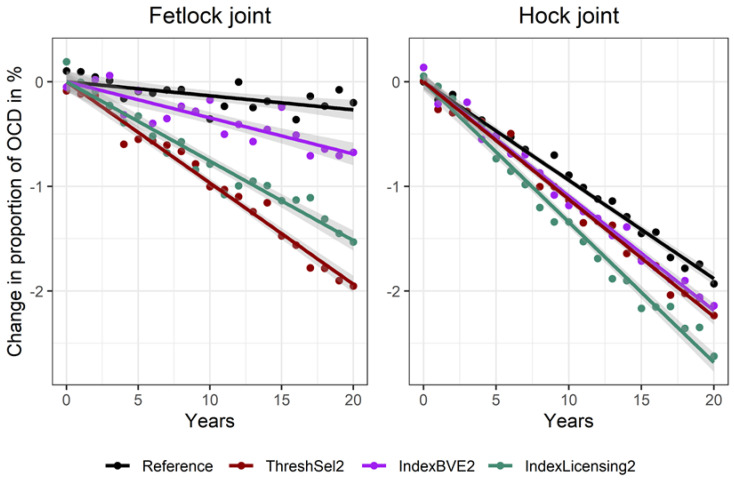
Proportion of unselected offspring with OCD in fetlock joints and hock joints for a reference scenario and for three different strategies for selection against OCD.

When the scenarios with the highest effect on the OCD traits from all considered scenario classes were compared, we observed a higher reduction of OCD for threshold selection (ThreshSel2) compared to the scenarios that were simply implementing OCD traits in the selection index for breeding stallions (IndexBVE2). However, when an additional selection step that accounted for OCD at the stage of stallion licensing (IndexLicensing2) was included, gains were very similar, with IndexLicensing2 leading to a slightly higher reduction in the hock joint and ThreshSel2 to a slightly higher reduction in the fetlock joint (Figure 5).

**Figure 6:**
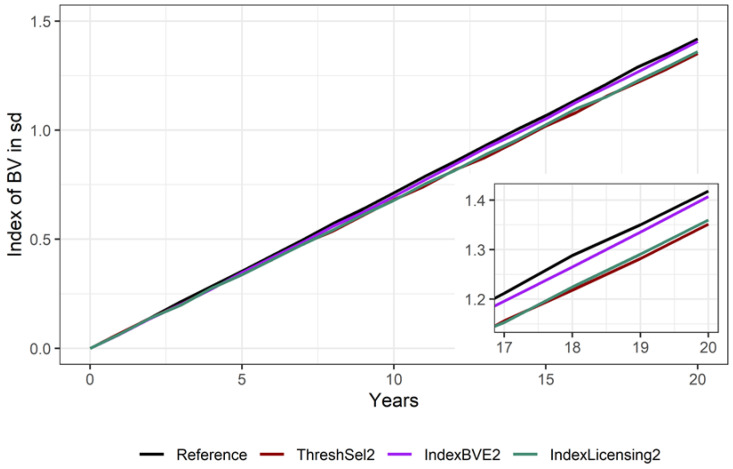
Average increase in breeding values in genetic standard deviations for the traits of the 50-day stallion performance test for scenarios without genomic breeding value estimation. The inset shows the period from 17 to 20 years.

Across all scenarios, the performance traits of the 50-day stallion performance test increased by an average of about 1.4 to 1.5 genetic standard deviations for the unselected offspring within 20 years. Scenarios ThreshSel1 and IndexLicensing2, which also scored rather equally in terms of OCD, also showed rather similar genetic gain for the performance traits. In comparison, the scenario IndexBVE2 almost lead to the same gains for the performance traits as the reference scenario (Figure 6). However, the difference between the most extreme scenarios (reference and ThreshSel2) was just 0.07 genetic standard deviations after 20 years.

**Figure 7:**
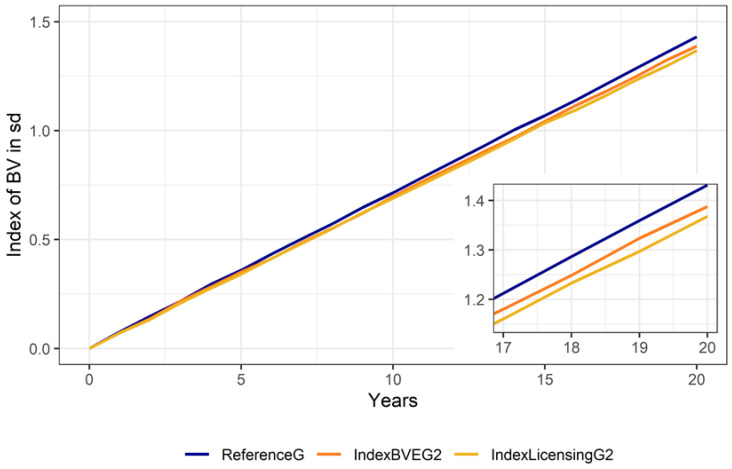
Average increase in breeding values in genetic standard deviations for the traits of the 50-day stallion performance test for scenarios with genomic breeding value estimation. The inset shows the period from 17 to 20 years.

Results for scenarios using genomic selection were similar, with a higher reduction of OCD frequency as in scenario IndexLicensingG2, leading to slightly lower gain (0.07 genomic standard deviations) for the riding horse performance traits (Figure 7). The increase in inbreeding in all scenarios was negligible with an average increase of the average kinship between 0.0033 and 0.0038 within 20 years and no clear pattern of differences between scenarios. The height at withers was only marginally influenced, with mean height at withers ranging between 166.7 cm (ThreshSel2) and 167.0 cm (ReferenceG) after 20 years.

## 4. Discussion

The study showed that even the reference scenario without selection against OCD led to a reduction of OCD in the hock joint whereas the frequency of OCD in the fetlock joint stayed rather constant. This was most likely due to a combination of a much higher heritability of OCD in the hock joint and the overall greater negative correlations to the performance traits. When using a threshold selection strategy, the strict threshold to not consider animals with OCD in any of the two joints for selection can be regarded as the preferable strategy in terms of reduction of OCD. Note, however, that this strategy led to the lowest genetic gain for riding horse performance traits. Only considering OCD in hock joint for selection led to an increase of OCD in fetlock joint. Therefore, it should be kept in mind that other traits like OCD in the distal interphalangeal joint and in the proximal interphalangeal joint were not considered in this study, as both have been shown to be negatively correlated with the trait OCD in hock joint (−0.341 and −0.666) [6]. Consequently, selecting for lower frequency of OCD in the hock joint may increase occurrence of OCD in the other joints. However, the same may be true for other traits with possibly unknown correlations to the characteristics under consideration. In contrast, some traits which would decrease along with OCD frequency in the fetlock joint, like deforming arthropathy in the hock joint, would be influenced favorably [22].

Plain integration of health traits in a selection index for breeding value estimation, which only influences the frequency of breeding use of stallions, performed substantially less effectively than additionally integrating these traits in a selection index at the stage of stallion licensing, which is the step with the highest selection intensity in the breeding program. This can be explained by the increase of the selection intensity for OCD when integrating OCD traits at the stage of stallion licensing. Note that in contrast to threshold selection, the success of OCD reduction can be controlled more flexibly through the adaptation of the relative index weight assigned to OCD traits. In a simulation study on the trait osteochondrosis (OC), Busche [23] found a higher genetic gain when using a breeding value estimation compared to threshold selection. The study, however, used a selection criterion based on X-ray classification and did not exclude animals with minor findings from breeding. Since the considered trait, the chosen selection criterion and the weighting of the traits are of considerable importance, these results do not contradict our observations.

Even though the simulations using genomic breeding value estimation did not show a clear advantage in absolute values, the difference to the reference scenario was slightly higher compared to the scenarios that were only using the pedigree based BLUP animal model. It should however be noted that with high prediction accuracy at earlier life stages, the use of genotyping could be beneficial to reduce the generation interval or to save some phenotyping costs. In case the generation interval could be shortened due to genomic breeding value estimation, the reduction of OCD could be higher per time unit (e.g., year) [24,25]. Whether a possible reduction of phenotyping costs also leads to a reduction in total costs highly depends of the ratio of genotyping to phenotyping costs and on the number of phenotyped and genotyped horses. Furthermore, genomic selection would also be beneficial to resolve issues related to pedigree errors. As the quality of the pedigree for German warmblood is expected to be high, pedigrees used for BVE were assumed to have no errors.

Increasing the number of observed phenotypes in the simulation led to relatively small effects on the frequency of OCD occurrence, whereas the additional integration of OCD-based selection on the stage of stallion licensing and the use of threshold selection for the inclusion of OCD in the breeding program had substantial impact on OCD frequency. As prediction accuracies were still improving with more available phenotypes, we would still recommend increasing phenotyping efforts. Note that clinical aspects of recording health traits are ignored in this simulation study and assumed to be the same across all scenarios. However, improving recording strategies is also a way of reducing OCD as we would expect reduced residual variances and thus higher heritability for health traits. Regarding the breeding progress in performance traits, slight differences between the scenarios were found, but none of the strategies for selection against OCD led to a major decline in the genetic progress for riding horse performance traits. Stock and Distl [22,26] also came to a similar conclusion in a study on the prediction of breeding values for different bone health traits. To further reduce OCD frequency, it might even be considered to further increase the index weights of the OCD features without risking a massive drop in performance traits. With regard to the height at withers, the correlation to the OCD traits led to no major effect with a difference between the extreme scenarios of less than 3 mm after 20 years. Furthermore, no substantial systematic increase of the average inbreeding was observed.

Regarding the implementation in practical breeding, it can be recommended to implement selection against OCD in fetlock joint and potentially even against OCD in both joints, as performance traits were only mildly affected and substantial improvements for the health traits were achieved. Note, however, that in addition to the OCD traits considered in this work other health traits, diseases and related clinical entities such as epiphysitis, subchondral cysts and angular carpal deformities [3] could also be considered as traits to select for. As OCD in the hock joint is improving without direct measures against it, one could focus on other traits instead. In the comparison of the different strategies the threshold selection is the simplest one for the practical use, indeed. A strict threshold selection was shown to be a valuable and competitive strategy, but the simulation also showed that approximately the same amount of reduction of OCD occurrence can be achieved with a proper integration of these traits in a selection index. This strategy could possibly have a higher acceptance of the breeders, especially the stallion owners, because there is no direct discrimination of individual stallions due to a single trait. Further benefit of the use of a selection index is that it provides a more flexible and dynamic way to adapt the weight put on OCD traits in the selection procedure. Additionally, selection based on indices and thus on a rather large number of relatives and not only on the phenotype of the stallion itself is ultimately less prone to fraud by clandestine surgeries.

Approaches combining the above discussed breeding strategies should also be considered. A pedigree-based breeding value estimation for OCD for mares would also be conceivable, provided a sufficient accuracy can be achieved through a high number of phenotyped animals. Finally, consideration should be given to an optimization of index weights, which could be based on economic considerations [27] as well as on desired outcomes for different phenotype categories [28].

## 5. Conclusions

Across all scenarios, the strongest effects on the frequency of OCD were observed in the scenario with the strict threshold selection and an index selection that was applied both at the stallion licensing and the determination of the frequency of breeding use. However, these scenarios also led to the highest loss in performance features, although the absolute changes for the performance traits were rather low. In this regard, it is important to consider the correlation of the individual health traits with the performance traits. It has been shown that the inclusion of OCD in the hock joint in the breeding scheme is not as important as in the fetlock joint, since genetic correlations already indirectly led to substantial improvement of OCD in the hock joint. As similar gains were obtained with both, threshold selection and the selection index at licensing, both methods are potential candidates for a practical use. One of the main advantages of a threshold selection is its relatively simple potential implementation. The implementation in a selection index is more complex but comes with the beneficial possibility of gradually shifting weight between health and performance traits.

## Figures and Tables

**Table 1:** Heritabilities (h^2^) and phenotypic standard deviations (σ_P_) of the considered performance traits for foal show [18], studbook registration [18], mare performance test [19], 14-day performance test [19] and 50-day performance test [19].

Event	Trait	h^2^	$\sigma_{P}$
Foal show	Type	0.50	0.79
	Exterior	0.50	0.62
	Movement	0.41	0.87
Studbook registration	Walk	0.25	0.80
	Trot	0.42	0.88
	Canter	0.49	0.71
Mare performance test	Walk	0.26	0.79
	Trot	0.40	0.76
	Canter	0.36	0.71
	Rideability	0.28	0.73
	Free-jumping	0.36	0.92
14-day performance test	Walk	0.41	0.81
	Trot	0.71	0.74
	Canter	0.56	0.60
	Rideability	0.50	0.65
	Free-jumping	0.74	0.71
50-day performance test	Walk	0.33	0.80
	Trot	0.47	0.85
	Canter	0.42	0.74
	Rideability	0.36	0.89
	Free-jumping	0.47	0.99
	Course jumping	0.40	1.12

**Table 2:** Heritabilities (diagonal), genetic correlations (above diagonal) and residual correlations (below diagonal) of osteochondrosis dissecans in fetlock joints (OCD-FJ), osteochondrosis dissecans in hock joints (OCD-HJ) and of the height at withers [6].

	OCD-FJ	OCD-HJ	Height at Withers
**OCD-FJ**	0.189	−0.27	0.349
**OCD-HJ**	0.085	0.369	0.181
**Height at withers**	0.037	0.119	0.275

**Table 3:** Additive genetic correlation between osteochondrosis dissecans (OCD) and selected performance traits for mare performance test. [7]

Trait	OCD-FJ	OCD-HJ
Walk	−0.01	−0.11
Trot	−0.04	−0.06
Canter	0.08	-0.09
Rideability	−0.03	−0.06
Free-jumping	−0.09	0.01

**Table 4:** Reason for trait recording and selection criteria with regard to OCD for seven different simulation scenarios.

Scenario	Trait Recording	Kind of OCD Selection	Selection Criterion OCD
Reference	None	None	No selection against OCD
ThreshSel1	Stallion licensing	Threshold selection	If fetlock and hock joints are affected
ThreshSel2			If fetlock or hock joints are affected
ThreshSelFJ			If fetlock joints are affected
ThreshSelHJ			If hock joints are affected
IndexBVE1	Stallion licensing	Index selection with effect on the frequency of breeding	OCD in fetlock and hock joints with the weight of one performance trait in the BVE
IndexBVE2	Stallion licensing and pre-purchase inspections ^1^		
IndexLicensing1	Stallion licensing	Additionally: Index selection at the stage of stallion licensing	OCD in fetlock and hock joints with the weight of one performance trait in the BVE
IndexLicensing2	Stallion licensing and pre-purchase inspections ^1^		

^1^ 50% of all horses.

**Table 5:** Accuracy of breeding value estimation for OCD in fetlock joint and OCD in hock joint.

	Accuracy of Breeding Value Estimation	
Scenario	OCD in Fetlock Joint	OCD in Hock Joint
IndexBVE1	0.34	0.35
IndexBVE2	0.53	0.47
IndexLicensing1	0.50	0.44
IndexLicensing2	0.52	0.47
IndexBVEG2	0.55	0.51
IndexLicensingG1	0.52	0.44
IndexLicensingG2	0.52	0.47

## Supplementary Materials

The following are available online at https://www.mdpi.com/2076-2615/10/7/1153/s1. Table S1: Genetic correlation (above diagonal) and phenotypic correlations (below diagonal) between traits for foal shows, studbook registration, mare performance test, 14-day performance test for stallions and 50-day performance test, File S1: BreedingProgramsMoBPS.zip.
