# Supplementary material for: Simulation Study on the Integration of Health Traits in Horse Breeding Programs
Source: Animals (Basel). 2020 Jul 7;10(7):1153. doi: 10.3390/ani10071153 (PMC7401664; doi:10.3390/ani10071153)
Supplement: Supplementary file 1 [file animals-10-01153-s001.zip › SupplementaryFiles/TableS1.docx]

**Table S1**. Genetic correlation (above diagonal) and phenotypic. correlations (below diagonal) between traits for foal shows, studbook registration, mare performance test, 14-day performance test for stallions and 50-day performance test [14,15].

|  | **Foal show** | | | **Studbook registration** | | | **Mare performance test** | | | | | **14-day performance test** | | | | | **50-day performance test** | | | | | |
| --- | --- | --- | --- | --- | --- | --- | --- | --- | --- | --- | --- | --- | --- | --- | --- | --- | --- | --- | --- | --- | --- | --- |
|  | **T** | **E** | **M** | **W** | **T** | **C** | **W** | **T** | **C** | **R** | **FJ** | **W** | **T** | **C** | **R** | **FJ** | **W** | **T** | **C** | **R** | **FJ** | **CJ** |
| **T** | 1 | 0.84 [14] | 0.56 [14] |  |  |  |  |  |  |  |  |  |  |  |  |  |  |  |  |  |  |  |
| **E** | 0.16 [14] | 1 | 0.56 [14] |  |  |  |  |  |  |  |  |  |  |  |  |  |  |  |  |  |  |  |
| **M** | 0.15 [14] | 0.08 [14] | 1 | 0.48 [14] | 0.77 [14] | 0.69 [14] | 0.46 [14] | 0.37 [14] | 0.45 [14] |  | 0.09 [14] |  |  |  |  |  |  |  |  |  |  |  |
| **W** |  |  |  | 1 | 0.61 [14] | 0.50 [14] | 0.95 [14] | 0.55 [14] | 0.62 [14] | 0.50 [14] | 0.05 [14] |  |  |  |  |  |  |  |  |  |  |  |
| **T** |  |  |  | 0.20 [14] | 1 | 0.65 [14] | 0.60 [14] | 0.69 [14] | 0.71 [14] | 0.53 [14] | 0.18 [14] |  |  |  |  |  |  |  |  |  |  |  |
| **C** |  |  |  | 0.17 [14] | 0.34 [14] | 1 | 0.44 [14] | 0.51 [14] | 0.83 [14] | 0.52 [14] | 0.50 [14] |  |  |  |  |  |  |  |  |  |  |  |
| **W** |  |  |  | 0.22 [14] | 0.13 [14] | 0.12 [14] | 1 | 0.61 [15] | 0.57 [15] | 0.62 [15] | 0.03 [15] | 0.77 [15] |  |  |  |  | 0.89 [15] |  |  |  |  |  |
| **T** |  |  |  | 0.12 [14] | 0.22 [14] | 0.22 [14] | 0.37 [15] | 1 | 0.81 [15] | 0.81 [15] | 0.09 [15] |  | 0.89 [15] |  |  |  |  | 0.89 [15] |  |  |  |  |
| **C** |  |  |  | 0.10 [14] | 0.18 [14] | 0.28 [14] | 0.36 [15] | 0.61 [15] | 1 | 0.80 [15] | 0.20 [15] |  |  | 0.89 [15] |  |  |  |  | 0.89 [15] |  |  |  |
| **R** |  |  |  | 0.06 [14] | 0.09 [14] | 0.15 [14] | 0.36 [15] | 0.50 [15] | 0.51 [15] | 1 | 0.12 [15] |  |  |  | 0.89 [15] |  |  |  |  | 0.94 [15] |  |  |
| **FJ** |  |  |  | 0.06 [14] | 0.07 [14] | 0.13 [14] | 0.06 [15] | 0.10 [15] | 0.15 [15] | 0.12 [15] | 1 |  |  |  |  | 0.88 [15] |  |  |  |  | 0.95 [15] |  |
| **W** |  |  |  |  |  |  | 0.25 [15] |  |  |  |  | 1 | 0.65 [15] | 0.55 [15] | 0.64 [15] | -0.31 [15] | 0.99 [15] |  |  |  |  |  |
| **T** |  |  |  |  |  |  |  | 0.33 [15] |  |  |  | 0.41 [15] | 1 | 0.79 [15] | 0.89 [15] | -0.32 [15] |  | 0.99 [15] |  |  |  |  |
| **C** |  |  |  |  |  |  |  |  | 0.40 [15] |  |  | 0.38 [15] | 0.68 [15] | 1 | 0.84 [15] | -0.18 [15] |  |  | 0.98 [15] |  |  |  |
| **R** |  |  |  |  |  |  |  |  |  | 0.32 [15] |  | 0.41 [15] | 0.65 [15] | 0.61 [15] | 1 | -0.35 [15] |  |  |  | 0.99 [15] |  |  |
| **FJ** |  |  |  |  |  |  |  |  |  |  | 0.39 [15] | -0.02 [15] | -0.05 [15] | 0.08 [15] | 0.02 [15] | 1 |  |  |  |  | 0.98 [15] |  |
| **W** |  |  |  |  |  |  | 0.26 [15] |  |  |  |  | 0.45 [15] |  |  |  |  | 1 | 0.72 [15] | 0.68 [15] | 0.65 [15] | -0.15 [15] | -0.05 [15] |
| **T** |  |  |  |  |  |  |  | 0.26 [15] |  |  |  |  | 0.66 [15] |  |  |  | 0.52 [15] | 1 | 0.81 [15] | 0.82 [15] | -0.06 [15] | 0.01 [15] |
| **C** |  |  |  |  |  |  |  |  | 0.34 [15] |  |  |  |  | 0.58 [15] |  |  | 0.51 [15] | 0.71 [15] | 1 | 0.80 [15] | 0.17 [15] | 0.21 [15] |
| **R** |  |  |  |  |  |  |  |  |  | 0.29 [15] |  |  |  |  | 0.52 [15] |  | 0.47 [15] | 0.66 [15] | 0.63 [15] | 1 | 0.03 [15] | 0.20 [15] |
| **FJ** |  |  |  |  |  |  |  |  |  |  | 0.31 [15] |  |  |  |  | 0.57 [15] | 0.11 [15] | 0.12 [15] | 0.20 [15] | 0.20 [15] | 1 | 0.93 [15] |
| **CJ** |  |  |  |  |  |  |  |  |  |  |  |  |  |  |  |  | 0.16 [15] | 0.19 [15] | 0.27 [15] | 0.33 [15] | 0.55 [15] | 1 |

T= Type, E = Exterior, M = Movement, W = Walk, T = Trot, C = Canter, R = Rideability, FJ = Free-jumping, CJ = Course jumping
